# Coronavirus disease 2019–related myocardial injury is associated with immune dysregulation in symptomatic patients with cardiac magnetic resonance imaging abnormalities

**DOI:** 10.1093/cvr/cvae159

**Published:** 2024-07-29

**Authors:** Andrej Ćorović, Xiaohui Zhao, Yuan Huang, Stephen R Newland, Deepa Gopalan, James Harrison, Despina Giakomidi, Shanna Chen, Natalia S Yarkoni, Christopher Wall, Marta Peverelli, Rouchelle Sriranjan, Arianna Gallo, Martin J Graves, Andrew Sage, Paul A Lyons, Nyarie Sithole, Martin R Bennett, James H F Rudd, Ziad Mallat, Tian X Zhao, Meritxell Nus, Jason M Tarkin

**Affiliations:** Section of Cardiorespiratory Medicine, Department of Medicine, University of Cambridge, Cambridge, UK; Section of Cardiorespiratory Medicine, Department of Medicine, University of Cambridge, Cambridge, UK; Section of Cardiorespiratory Medicine, Department of Medicine, University of Cambridge, Cambridge, UK; Section of Cardiorespiratory Medicine, Department of Medicine, University of Cambridge, Cambridge, UK; Department of Radiology, Cambridge University Hospitals NHS Trust, Cambridge, UK; Section of Cardiorespiratory Medicine, Department of Medicine, University of Cambridge, Cambridge, UK; Section of Cardiorespiratory Medicine, Department of Medicine, University of Cambridge, Cambridge, UK; Section of Cardiorespiratory Medicine, Department of Medicine, University of Cambridge, Cambridge, UK; Cell Phenotyping Hub, Department of Medicine, University of Cambridge, Cambridge, UK; Section of Cardiorespiratory Medicine, Department of Medicine, University of Cambridge, Cambridge, UK; Section of Cardiorespiratory Medicine, Department of Medicine, University of Cambridge, Cambridge, UK; Section of Cardiorespiratory Medicine, Department of Medicine, University of Cambridge, Cambridge, UK; Section of Cardiorespiratory Medicine, Department of Medicine, University of Cambridge, Cambridge, UK; Department of Radiology, University of Cambridge, Cambridge, UK; Section of Cardiorespiratory Medicine, Department of Medicine, University of Cambridge, Cambridge, UK; Cambridge Institute of Therapeutic Immunology and Infectious Disease, Jeffrey Cheah Biomedical Centre, Cambridge Biomedical Campus, Cambridge, UK; Department of Medicine, University of Cambridge, Cambridge, UK; Infectious Diseases, Department of Medicine, University of Cambridge, Cambridge, UK; Section of Cardiorespiratory Medicine, Department of Medicine, University of Cambridge, Cambridge, UK; Section of Cardiorespiratory Medicine, Department of Medicine, University of Cambridge, Cambridge, UK; Section of Cardiorespiratory Medicine, Department of Medicine, University of Cambridge, Cambridge, UK; Section of Cardiorespiratory Medicine, Department of Medicine, University of Cambridge, Cambridge, UK; Section of Cardiorespiratory Medicine, Department of Medicine, University of Cambridge, Cambridge, UK; Section of Cardiorespiratory Medicine, Department of Medicine, University of Cambridge, Cambridge, UK

**Keywords:** COVID-19, Magnetic resonance imaging, Immunophenotyping, Myocarditis

## Abstract

**Aims:**

While acute cardiovascular complications of coronavirus disease 2019 (COVID-19) are well described, less is known about longer-term cardiac sequelae. For many individuals with cardiac signs or symptoms arising after COVID-19 infection, the aetiology remains unclear. We examined immune profiles associated with magnetic resonance imaging (MRI) abnormalities in patients with unexplained cardiac injury after COVID-19.

**Methods and results:**

Twenty-one participants {mean age 47 [standard deviation (SD) 13] years, 71% female} with long COVID-19 (*n* = 17), raised troponin (*n* = 2), or unexplained new-onset heart failure (*n* = 2), who did not have pre-existing heart conditions or recent steroid/immunosuppression treatment, were enrolled a mean 346 (SD 191) days after COVID-19 infection in a prospective observational study. Cardiac MRI and blood sampling for deep immunophenotyping using mass cytometry by time of flight and measurement of proteomic inflammatory markers were performed. Nine of the 21 (43%) participants had MRI abnormalities (MRI(+)), including non-ischaemic patterns of late gadolinium enhancement and/or visually overt myocardial oedema in 8 people. One patient had mildly impaired biventricular function without fibrosis or oedema, and two had severe left ventricular (LV) impairment. MRI(+) individuals had higher blood CCL3, CCL7, FGF-23, and CD4 Th2 cells, and lower CD8 T effector memory (TEM) cells, than MRI(−). Cluster analysis revealed lower expression of inhibitory receptors PD1 and TIM3 in CD8 TEM cells from MRI(+) patients than MRI(−) patients, and functional studies of CD8 T αβ cells showed higher proportions of cytotoxic granzyme B^+^(GZB^+^)-secreting cells upon stimulation. CD8 TEM cells and CCL7 were the strongest predictors of MRI abnormalities in a least absolute shrinkage and selection operator regression model (composite area under the curve 0.96, 95% confidence interval 0.88–1.0). CCL7 was correlated with diffuse myocardial fibrosis/oedema detected by quantitative T1 mapping (*r* = 0.47, *P* = 0.04).

**Conclusion:**

COVID-19-related cardiac injury in symptomatic patients with non-ischaemic myocarditis-like MRI abnormalities is associated with immune dysregulation, including decreased peripheral CD8 TEM cells and increased CCL7, persisting long after the initial infection.

## Graphical Abstract

**Graphical Abstract:**
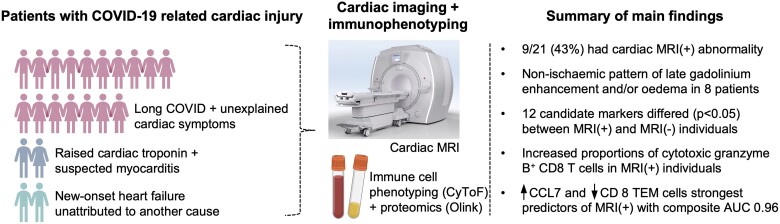
Summary of study methods and main findings.

**Time of primary review: 22 days**

## 1. Introduction

Cardiovascular involvement in coronavirus disease 2019 (COVID-19) encompasses a wide range of vascular and myocardial pathologies, including both acute and long-term sequelae.^1^ Myocardial injury inferred by elevated cardiac troponin (cTn) is not uncommon in COVID-19 infection, having been reported in 20–40% of hospitalized patients at the start of the pandemic.^2,3^ In this setting, cTn elevation is associated with the severity of COVID-19 infection and provides an independent prognostic marker,^4,5^ either as cause or consequence of a stormy clinical course. Conversely, cardiac involvement in post-acute sequelae of severe acute respiratory syndrome coronavirus 2 (SARS-CoV-2) infection (or ‘long COVID-19’) tends not to be related to the severity of the initial infection and remains less well understood.^6,7^

Depending on the clinical scenario, a cTn leak in patients with active COVID-19 could either signify direct cardiac involvement in COVID-19 or serve as a non-specific marker of a severe systemic insult with the heart as a bystander. Viral myocarditis is among the many causes of cardiac injury that has been described in patients with COVID-19; however, acute myocarditis appears to occur more rarely than initially suspected with an overall low prevalence of 2.4 per 1000 hospitalized patients.^8^ Other mechanisms of cTn elevation in patients with COVID-19 include acute myocardial infarction (MI) due to atherosclerotic plaque rupture or demand ischaemia driven by a systemic inflammatory state; endothelial and microvascular dysfunction; immune-mediated activation of coagulation and fibrinolytic systems leading to thrombo-embolism; heart failure, cardiogenic shock, tachyarrhythmias, and stress cardiomyopathy.^9,10^ Importantly, an early case series showed that a high proportion of patients with COVID-19 presenting with chest pain and electrocardiograms (ECGs) showing ST-segment elevation did not have obstructive coronary disease on angiography, demonstrating the often-murky underlying aetiology.^11^ Indeed, an autopsy study of 40 hearts from hospitalized patients who died of COVID-19 showed that while myocyte necrosis was present in 35% of the hearts, most patients did not suffer from an acute MI, and small vessel microthrombi were observed far more often than epicardial artery thrombi.^12^ A literature review of 227 post-mortem reports from 22 studies also found at least one COVID-19-related cardiovascular finding in 48% of cases, with myocarditis in <2%, but no distinct histopathological description of COVID-19 myocardial injury.^13^

In efforts to better characterize the clinical manifestations of cardiac involvement in patients with COVID-19, imaging has been utilized in several large cohort studies. In a multinational registry study of echocardiography performed in patients with COVID-19, findings to support a new MI or myocarditis diagnosis were found in just 3% of 1216 patients.^14^ However, in a case-control study of 58 patients assessed 2–3 months after COVID-19 infection requiring hospitalization, native myocardial T1 mapping values and %late gadolinium enhancement (LGE) indicating myocardial fibrosis on cardiac magnetic resonance imaging (MRI) were increased compared with controls, despite cTn elevation occurring in only 8% of individuals at the time of the illness.^15^ T1 mapping is the longitudinal relaxation time of tissues per voxel, which is affected by the composition of intracellular and extracellular compartments (i.e. protein, water, lipid, and iron content). An elevated T1 is reflective of interstitial fibrosis or oedema in the absence of an infiltrative cardiomyopathy. A larger study of 342 hospitalized patients with COVID-19 and raised cTn imaged within 28 days of discharge revealed a two-fold greater incidence of cardiac abnormalities compared to matched controls, with a higher burden of left and right systolic impairment, pericardial effusions, and scar from infarction and microinfarction.^16^ Other investigations of cardiac MRI findings in similar patient cohorts identified higher incidences of myocarditis-related abnormalities.^17,18^ Elevated T2 mapping values on MRI suggestive of diffuse myocardial oedema have also been reported in patients with persistent long-term cardiac symptoms after a mild COVID-19 illness who had no previous cardiac history or cTn elevation.^19^ Moreover, signs and symptoms of autonomic dysfunction in the absence of structural cardiac abnormalities also develop in some patients after COVID-19.^20^ However, much remains to be known about the underlying mechanisms driving cardiovascular involvement in COVID-19.

Dysregulation of the immune system and defective immune recovery have been implicated as key players in determining both the severity of acute COVID-19 infection and predisposing to longer-term symptoms. The kinetics of immune changes in COVID-19 were investigated in serial blood samples from 207 SARS-CoV-2-infected individuals with a range of disease severities over 12 weeks from symptom onset.^21^ That study showed that mild/asymptomatic infection was associated with a robust adaptive immune cell response, with early rise and fall of effector CD8^+^ T cells and circulating plasmablasts, as well as transient complement activation, but no other evidence of systemic inflammation. Conversely, patients with severe infection failed to mount early B and T cell responses and had persistently elevated inflammatory markers [C-reactive protein, interleukin 6 (IL-6), and tumour necrosis factor alpha (TNF-α)]. Various proteomic immune signatures associated with clinical outcomes after COVID-19 have also been reported.^22,23^ Moreover, studies of patients with long COVID-19 have identified key immunological differences relative to matched control populations for up to 13 months, including altered subsets of T and B cells, monocytes, dendritic cells (DCs), PD1 expression, and interferon responses.^24–26^

However, the specific immune responses associated with cardiac complications arising after COVID-19 infection remain unknown. A better understanding of the immune mechanisms that contribute to the increased morbidity and mortality risk, and impaired quality of life, caused by COVID-19-related myocardial injury is needed to inform future translational research and improve clinical management. By integrating clinical imaging and deep immune phenotyping in a prospective proof-of-concept study, we aimed to test the hypothesis that patients with both clinical and MRI evidence of cardiac involvement in COVID-19 would have distinct alterations of their systemic immune profiles compared to those without.

## 2. Methods

### 2.1 Study design and participants

Male and female participants over 18 years old with a history of COVID-19 infection and suspected cardiac involvement were enrolled in a prospective observational study between October 2020 and February 2022 at Cambridge University Hospitals NHS Foundation Trust, Cambridge, UK [the Multimodality Imaging and Immunophenotyping of COVID-19-related Myocardial Injury (MIIC-MI) study; ClinicalTrials.gov: NCT04412369). Patients with clinically suspected cardiac involvement after COVID-19 infection were included from three categories: (i) long COVID-19 with persistent, unexplained cardiac symptoms; (ii) non-specified cTn I elevation > 99th percentile of upper reference limit; or (iii) recent-onset heart failure unattributed to another cause. Participants were identified from hospital inpatient wards or outpatient clinics and the Cambridge National Institute for Health and Care Research (NIHR) BioResource. Long COVID-19 was diagnosed as per clinical criteria,^27^ independent of the study findings.

Participants with a prior history of MI, heart failure, or other pre-existing cardiac conditions unrelated to COVID-19 were excluded, as were those with a high pre-test probability of an acute MI in the context of a recent COVID-19 infection. Patients who had received steroids or other systemic immunosuppressive agents within 1 month were also excluded because of the direct effects of these therapies on the immune responses being studied (see Supplementary material online, *Methods*, for full inclusion/exclusion criteria). Research was conducted in accordance with the protocol approved by the local research ethic committee (20/SW/0090) and the Declaration of Helsinki. All participants gave written informed consent prior to inclusion. The retention, storage, and use of blood samples were subject to the UK Human Tissue Act 2004.

### 2.2 Imaging

Cardiac MRI was performed on a 1.5 Tesla scanner (SIGNA Artist, GE Healthcare, USA) following a standard clinical protocol. MRI acquisitions included axial breath-held proton-density weighted, blood-suppressed single-shot fast-spin echo imaging (8 mm slice thickness); steady-state free precession cine imaging of the ventricles; T2-weighted turbo spin echo with short-inversion-time-inversion-recovery (STIR) and blood suppression; T1 mapping using modified look-locker inversion recovery; fast imaging employing steady-state acquisition; T2 mapping using blood-supressed fast-spin echo; and early gadolinium enhancement and LGE.

Participants were stratified into two groups based on MRI findings: ‘MRI(+)’ and ‘MRI(−)’. The pre-defined criteria for MRI(+) was one or more of the following cardiac abnormalities: impaired left- or right-sided cardiac function (volumetric ejection fraction below 2 SDs from the mean for age and sex^28^) ± dilated ventricular size (indexed to body surface area); focal myocardial oedema (visually overt high signal on T2-weighted imaging ± quantitative T2 mapping above the reference range); LGE (sub-endocardial, mid-wall, sub-epicardial, or transmural LGE); and pericardial enhancement and/or effusion. Those with borderline/‘soft’ MRI abnormalities such as isolated T1/T2 mapping or global longitudinal strain values outside the normal reference range or mild insertion site fibrosis were not included in the MRI(+) group.

For patients with a clinical indication for coronary computed tomography angiography (CCTA), scans were performed using a dual-source scanner (SOMATOM Definition Flash, Siemens, Germany), following a standard clinical protocol that included pre-medication with sublingual glyceryl trinitrate ± intravenous metoprolol. Contrast-enhanced ECG-gated CCTA images were acquired during end-inspiration and reconstructed at 0.75 mm slice thickness.

### 2.3 Image analysis

Cardiac MRI analysis was performed on a clinical workstation using dedicated imaging software (cvi42, Circle Cardiovascular Imaging Inc., Canada). Clinical reporting was conducted by experienced cardiac MRI readers, independent of the study. Additional quantitative analyses were also undertaken with the reader blinded to the study findings. MRI analyses included assessment of cardiac chamber size (absolute and indexed volumes) and function (ejection fraction and longitudinal strain), wall thickness, ventricular mass, wall motion, valve function, myocardial oedema, quantitative T1/T2 mapping, the pattern and distribution of LGE, and the pericardium. CCTAs were evaluated independently of the study by an experienced reporter on a clinical workstation (Syngo.via, Siemens, Germany), as per usual clinical care.

### 2.4 Immune cell phenotyping

Venous blood samples collected in 6 mL heparinized tubes were taken at the time of imaging and analysed using mass cytometry by time of flight (CyTOF). CyTOF uses metal isotopes to identify and tag extracellular molecules, separates single cells by flow cytometry, and simultaneously quantifies multiple labelled cellular components using inductively coupled plasma mass spectrometry based on the atomic mass spectrum of single cells. Briefly, an aliquot of 250 μL of fresh heparinized blood was stained with a panel of 30 metal conjugated antibodies using the Maxpar Direct Immune Profiling Assay (Standard BioTools Inc., USA). PD1-175Lu, TIM3-159Tb, and PDL1-169Tm (Standard BioTools Inc., USA) were also added to the panel. Staining was performed as per the manufacturer's specification to identify immune cell subsets that were gated manually following the strategy described in Supplementary material online, *Table S1*. Stained blood samples were cryopreserved in liquid nitrogen and stored until being run in batches of 7 on a Helios system. Results were analysed initially using Maxpar Pathsetter software, FlowJo, and FCS Express (De Novo Software). CyTOF results are displayed as percentage (%) of total cells for main cell types or parent cells for subtypes.

### 2.5 Intracellular staining and functional assays

Frozen peripheral blood mononuclear cell (PBMC) aliquots were thawed, and cells cultured for 6 h with Leukocyte Activation Cocktail (phorbol 12-myristate 13-acetate, phorbol 12, 13-dibutyrate, and ionomycin) with BD GolgiPlug containing brefeldin A (BD Biosciences, USA). Then, samples were stained with extracellular markers (1:200) for 30 min before intracellular fixation buffer (eBioscience, USA) for another 30 min. Cells were washed with fix/perm solution (eBioscience, USA) and stained with intracellular antibodies (1:800) overnight as previously described (see Supplementary material online, *Table S2*, for antibodies used).^29^ Samples were analysed using a Cytek Aurora spectral flow cytometer following the manufacturer instructions.

### 2.6 Proteomic analysis

Blood samples collected in serum separator tubes were centrifuged at 4000 *g* for 7 min and stored at −80°C. Stored serum was thawed and 40 μL aliquoted and randomized on a single 96-well plate and shipped to Olink (Uppsala, Sweden) for proteomic analysis using the Target 96 inflammation panel. This analysis uses Proximity Extension Assay technology, which combines antibody-based immunoassays with real-time quantitative polymerase chain reaction (PCR) to enable high-throughput, multiplex immunoassays of proteins.

### 2.7 Statistical methods

Statistical analyses were performed in R (version 4.2.1, R Core Team). To compare peripheral blood immune cell phenotyping (CyTOF) and proteomic data (Olink) stratified by MRI findings, data were transformed using the Yeo–Johnson transform, followed by scaling and centring using ‘tidymodels’, and visualized using heatmaps generated by ‘ComplexHeatmap’.^30^ Mean transformed data were compared between MRI(+) and MRI(−) individuals using the Student's *t* test and visualized in a volcano plot. To visualize the clustering of features contributing to the inter-group difference, principal component analysis was conducted on candidate features with *P* < 0.1, with graphs generated using ‘factoextra’.

Cluster analyses were performed using raw flow cytometry data files and the R packages ‘flowCore’ and ‘Seurat’.^31,32^ After individual data clustering, a harmony method was applied for batch correction and integration analysis across all patients with down sampling 300 cells in each cluster. A total of 34 markers and 97 013 cells were input for the downstream analysis. Dimension reduction with resolution value 1.2 was used to optimize clusters, which were manually annotated, and data displayed in UMAPs and dot plots for all cells and T cell sub-clusters. Differential expression analysis between MRI(+) and MRI(−) groups was performed using Seurat ‘FindMarkers’ function with ‘Wilcox’ test and selected markers (adj.*P* < 0.05) displayed in dot plots. Annotated cell proportion test between MRI(+) and MRI(−) groups used scProportionTest, with results shown in metaplot.

For differentially expressed proteins, unsupervised pathway analysis was performed using STRING to summarize the network of predicted associations.^33^ STRING gene set enrichment analysis was also performed for tissues, processes, and WikiPathways.

All blood markers and baseline clinical data were then incorporated into a multivariable logistic regression model with MRI(+) as the outcome. To avoid potential overfitting, least absolute shrinkage and selection operator (LASSO) regularization was used to identify the most clinically relevant and robust predictive features. The hyperparameter *λ* in the LASSO algorithm was determined with leave-one-out cross validation. The performance of the model was evaluated by the receiver operating characteristic (ROC) using package ‘pROC’, and the area under the curve (AUC) reported. To explore the associations among blood markers with *P* < 0.05 and quantitative MRI mapping values, a correlation plot was generated using package ‘corrplot’, with the correlation matrix ordered by hierarchical clustering. Scatter plots were generated using Prism (version 9.1.0, GraphPad Software).

## 3. Results

### 3.1 Clinical study

Of the total 78 individuals screened, 21 participants {mean age 47 [standard deviation (SD) 13] years, 71% female} were enrolled who underwent imaging and blood sampling as per the study protocol (see Supplementary material online, *Figure S1*). Participants had a history of COVID-19 and persistent unexplained cardiac symptoms attributed to long COVID-19 (*n* = 17), raised cTn I due to suspected myocarditis (*n* = 2), or heart failure occurring after COVID-19 with no other cause identified (*n* = 2). Beyond medical history, COVID-19 infection was confirmed by either a positive result on PCR testing at the time of their initial infection and/or subsequent serology and/or T cell response assay (IL-2) to SARS-CoV-2 peptides performed as part of another study^34^ in 16 of the 17 patients who were tested. Although one long COVID-19 patient had a negative IL-2 T cell response despite fulfilling clinical criteria, this could be explained by the limited sensitivity of the assay.

Baseline clinical characteristics are summarized in *Table 1*. All patients had symptoms typical of COVID-19 at the time of their initial infection. Approximately half of the patients either attended or were admitted to hospital because of their symptoms related to COVID-19, but none required management on intensive care. Four of the 21 (19%) patients had abnormalities (conduction system delay or non-specific T wave changes) identified on resting ECGs that were not previously known. There were no sustained arrhythmias identified on ambulatory electrocardiography recordings in any of the nine patients with a clinical indication for this investigation.

**Table 1:** Baseline clinical characteristics

	MRI (+)	MRI (−)
Participants (*n*)	9	12
Mean age, years (SD)	47 (16)	47 (11)
%Female (*n*)	67 (6)	75 (9)
Mean body mass index, kg/m^2^ (SD)	28 (4)	27 (8)
Mean days from symptoms to MRI (SD)	263 (167)	407 (191)
COVID-19 symptoms		
%Cough (*n*)	56 (5)	75 (9)
%Fever (*n*)	78 (7)	92 (11)
%Change in smell or taste (*n*)	33 (3)	58 (7)
%Hospital attendance or admission	56 (5)	50 (6)
Cardiac symptoms		
%Chest pain (*n*)	67 (6)	58 (7)
%Palpitations (*n*)	44 (4)	58 (7)
%Breathlessness (*n*)	89 (8)	75 (9)
%Pedal oedema (*n*)	11 (1)	0 (0)
Past medical history		
%Systemic hypertension (*n*)	33 (3)	0 (0)
%Hypercholesterolaemia (*n*)	22 (2)	0 (0)
%Diabetes mellitus (*n*)	0 (0)	0 (0)
%Autoimmune disorder (*n*)	11 (1)	17 (2)
%Family history of CVD ≤ 60 years (*n*)	11 (1)	33 (4)
%Current or ex-smoker (*n*)	78 (7)	8 (1)
Medications		
%Aspirin (*n*)	22 (2)	8 (1)
%Statin (*n*)	33 (3)	8 (1)
%ACE inhibitor or angiotensin receptor blocker (*n*)	44 (4)	0 (0)
%Beta-adrenergic receptor blocker (*n*)	56 (5)	25 (3)
%Mineralocorticoid receptor antagonist (*n*)	22 (2)	0 (0)
%Dapagliflozin (*n*)	11 (1)	0 (0)
%Colchicine (*n*)	33 (3)	0 (0)
%Non-steroidal anti-inflammatory (*n*)	22 (2)	8 (1)
Blood investigations		
Mean troponin I, ng/L (SD)	11.9 (15.7)	8.3 (8.6)
Mean NT-proBNP, pg/mL (SD)	739 (1013)	45 (33)
Mean high-sensitivity C-reactive protein, mg/L (SD)	6.6 (12.9)	1.7 (2.4)
Mean erythrocyte sedimentation rate, mm (SD)	8.8 (3.3)	7.9 (4.8)
Electrocardiography		
%PR-segment prolongation (*n*)	8.3 (1)	0 (0)
%Left bundle branch block (*n*)	16.7 (2)	0 (0)
%T wave abnormalities (*n*)	8.3 (1)	8.3 (1)

There was a mean 346 days (SD 191) from COVID-19 symptoms to MRI/blood sampling across the whole cohort. Three of the 21 (14%) patients were vaccinated for SARS-CoV-2 before their index COVID-19 infection; 13/21 (62%) were vaccinated after the infection but before imaging/blood sampling; and 4/21 (19%) were vaccinated after infection and after imaging/blood sampling. Of those who were vaccinated before infection or imaging/blood sampling, 11/16 (68%) received the AstraZeneca (Oxford) formulation as their first vaccine and the remaining 5 patients (31%) had Pfizer/BioNTech (Comirnaty). One of the patients enrolled with suspected myocarditis in the context of a recent COVID-19 infection also had a previous history of myocarditis 9 months earlier after receiving the COVID-19 vaccination. No other patients had suspected vaccine-induced cardiac reactions. There was a mean 97 days (SD 55) between the last vaccine dose and MRI/blood sampling among the 16 vaccinated patients who received their first dose before enrolment.

### 3.2 Imaging findings

**Figure 1:**
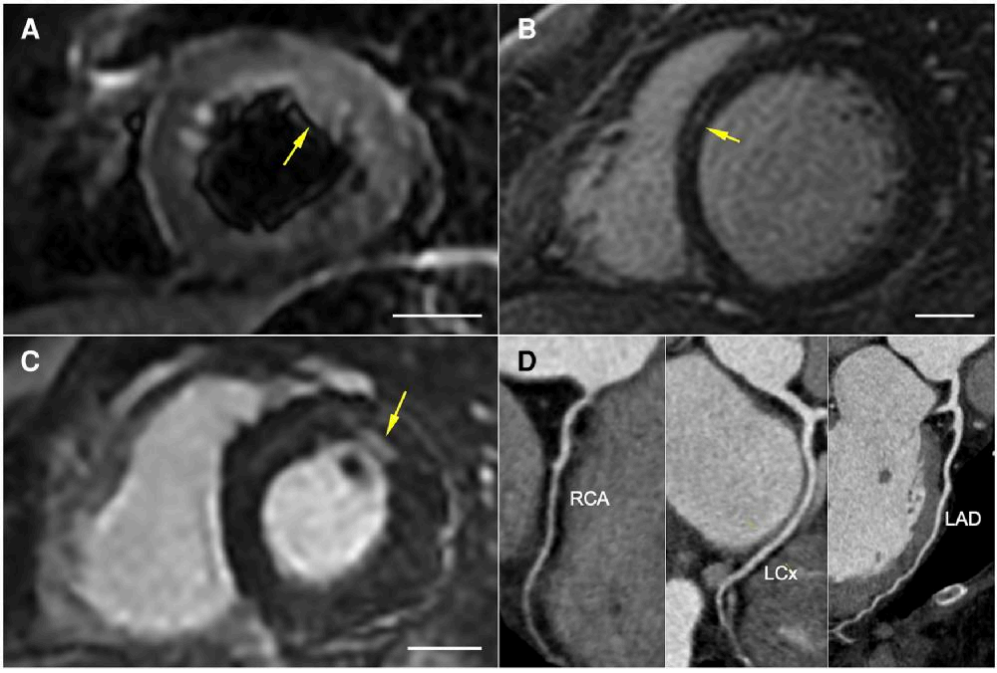
Cardiac MRI abnormalities in patients after COVID-19. Representative images showing cardiac MRI abnormalities (arrows) detected in patients after COVID-19 infection: (*A*) increased T2-weighted oedema signal in the mid-anterolateral segment; (*B*) linear mid-wall LGE in the basal septum; and a (*C*) small focus of basal anterior sub-endocardial LGE in a patient with (*D*) no epicardial coronary artery disease on CTA. Scale bar = 20 mm.

Abnormal cardiac MRI appearances were identified in 9/21 (43%) participants (*Figure 1* and *Table 2*). MRI abnormalities included impaired left- or right-sided cardiac function in 3/21 (14.3%), focal oedema in 2/21 (9.5%), and myocardial LGE in 8/21 (33.3%). These nine participants were classed as MRI(+), and the remaining were MRI(−). One patient did not have T2-weighted imaging, myomaps, or early/late gadolinium imaging performed due to a scanner malfunction. The myocardial distribution of LGE was mid-myocardial or mixed sub-epicardial/mid-myocardial in all but one patient. That participant had a small focus of sub-endocardial enhancement, but CCTA showed smooth and unobstructed coronary arteries supporting a non-ischaemic cause. Another patient with non-specific insertion site fibrosis did not fulfil pre-specified criteria for inclusion in the MRI(+) group. Overall, 2/21 (4.8%) patients fulfilled modified Lake Louise criteria for active myocarditis (one had suspected myocarditis and the other long COVID-19).^35^ Another participant had a small pericardial effusion. None of the participants had pericardial enhancement, microvascular obstruction, or *in situ* thrombus. While three patients were found to have mild coronary artery disease, none of the eight patients who underwent CCTA or invasive angiography as part of clinical care had obstructive coronary disease.

**Table 2:** MRI findings in patients with cardiac symptoms after COVID-19

Group	MRI (+)	Age (years)	Sex	Chamber size and function								Tissue characterization			
				LVEF (%)	LV EDV (mL)	LV EDVi (mL/m^2^)	RVEF (%)	RV EDV (mL)	RV EDVi (mL/m^2^)	GLS (%)	Oedema	T1 value (ms)	T2 value (ms)	LGE	Effusion
Long COVID-19	No	57	M	73	152	77	60	135	69	−17.6	No	978	48	No	No
Long COVID-19	No	28	F	62	132	80	61	133	81	−17.3	No	–	–	No	No
Long COVID-19	No	49	F	64	106	69	58	101	66	−19.1	No	998	39	No	No
Long COVID-19	No	44	M	66	123	53	58	147	64	−12.8	No	973	43	No	No
Long COVID-19	No	47	F	62	130	65	62	140	71	−17	No	1010	48	No	No
Long COVID-19	No	57	F	75	102	63	61	95	59	−18.8	No	958	49	No	No
Long COVID-19	No	34	F	65	116	67	60	126	72	−16.7	No	1016	51	No	No
Long COVID-19	No	66	F	68	79	46	62	87	51	−18.4	No	986	50	No	No
Long COVID-19	No	46	F	66	98	60	67	93	57	−18.4	No	893	46	No	No
Long COVID-19	No	36	M	56	160	77	56	132	64	−15.6	No	1005	50	No	No
Long COVID-19	No	41	F	56	134	73	58	125	68	−15.5	No	993	50	No	No
Long COVID-19	No	53	F	64	146	69	–	–	–	–	No	1020	48	No	No
Long COVID-19	Yes	55	F	61	160	80	57	139	70	−18.7	No	1016	53	Yes	No
Long COVID-19	Yes	54	F	67	129	73	61	147	83	−19.3	No	968	51	Yes	Yes
Long COVID-19	Yes	73	F	69	122	63	62	97	50	−15.5	Yes	1138	60	No	No
Long COVID-19	Yes	51	F	63	123	66	64	117	63	−18.1	No	1028	47	Yes	No
Long COVID-19	Yes	25	F	63	129	61	62	125	59	−16.4	No	1132	51	Yes	No
Raised cTn I	Yes	49	F	68	94	54	72	83	47	−20.9	Yes	1071	60	Yes	No
Raised cTn I	Yes	21	M	55	163	88	53	165	90	−15.1	No	960	54	No	No
Heart failure	Yes	57	M	20	399	188	52	146	69	−4.9	No	906	49	Yes	No
Heart failure	Yes	42	M	16	408	164	55	164	66	−3.9	No	1134	49	No	No

Normal ranges for T2: 50–57 ms and T1: 950–1060 ms.

cTn I, cardiac troponin I; GLS, global longitudinal strain; LGE, late gadolinium enhancement.

All patients with long COVID-19 had normal biventricular size and systolic function, but 4/17 (23.5%) had impaired global longitudinal strain above −16%, 1/17 (5.9%) had focal oedema on T2-weighted STIR imaging, and 4/17 (23.5%) had myocardial LGE. One of the two patients enrolled because of suspected myocarditis had borderline/mild impairment in left and right ventricular ejection fraction of 50–55% and reduced global longitudinal strain on MRI performed 116 days from symptom onset. That patient presented initially with chest pain, a normal ECG, peak cTn I 2511 ng/L (NR 0 to 58), and an echocardiogram at the time of initial presentation with mild-to-moderately reduced LV ejection fraction of 43%. The other patient with a clinical diagnosis of suspected myocarditis had focal oedema and mid-wall LGE confirmed on MRI. The two patients with new-onset heart failure after COVID-19 had severely reduced LV ejection below 20%. Both individuals had globally impaired and dilated left ventricles, with mild right ventricular impairment, normal wall thickness, normal T1/T2 relaxation times, and non-ischaemic patterns of LGE.

### 3.3 Follow-up imaging

Repeat MRI, performed after 111 and 90 days in the two patients with evidence of active myocarditis, showed inflammation resolution in both cases. There were no other follow-up MRIs performed as part of routine clinical care. The two patients with severe heart failure had improvements in LV ejection fraction following medical therapy, from below 20% to between 30–35% and 40–45% on repeat echocardiography after 2.4 and 1.4 years, respectively.

### 3.4 Immunophenotyping data

**Figure 2:**
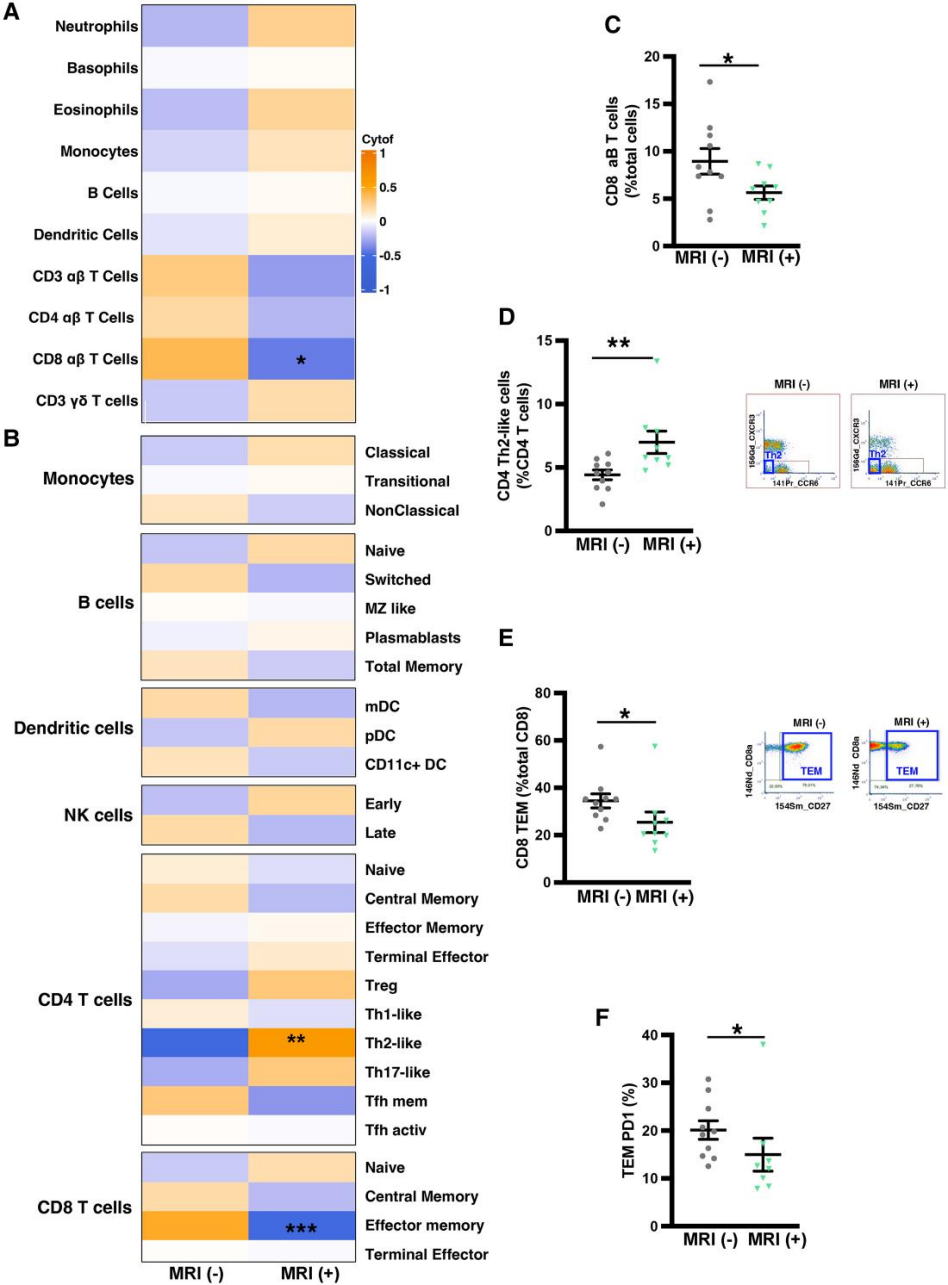
Deep immune cell phenotyping in patients stratified by cardiac MRI. (*A* and *B*) Heatmaps comparing circulating immune cell subsets in patients (*n* = 19) with (+) and without (−) cardiac MRI abnormalities. Transformed data displayed in heatmaps compared using Student's *t* test. Scatter dot plots with representative flow cytometry graphs showing proportions of (*C*) CD8 αβ T cells, (*D*) CD4 Th2-like cells, and (*E*) CD8 TEM and (*F*) CD8 PD1^+^ TEM cells in patients (*n* = 19) with (+) and without (−) MRI abnormalities compared using Student's *t* test; *P < 0.05; **P < 0.01; ***P < 0.001.

Analysis of PBMC populations revealed key differences in circulating immune cell subsets among patients with and without clinically detectable MRI abnormalities. There were insufficient cells isolated from two patients for CyTOF analysis. Three main cell subsets differed between the groups: CD8 αβ T cells, CD8 T effector memory (TEM), and CD4 Th2-like cells (*Table 3*). CD8 T αβ and CD8 TEM cells were lower in MRI(+) than MRI(−) patients (*Figure 2A–C* and *E*). In contrast, CD4 Th2-like cells were higher in MRI(+) than MRI(−) patients (*Figure 2B* and *D*). There were no differences detected in the proportions of granulocytes (neutrophils, basophils, and eosinophils), monocytes, DCs, B cells, or in other T cell subsets between MRI(+) and MRI(−) groups (*Figure 2A* and *B*).

**Table 3:** Differential markers for patients stratified by cardiac MRI

	*P*	Transformed		Raw	
		MRI(−)	MRI(+)	MRI(−)	MRI(+)
CCL7	0.001	−0.535	0.849	1.757	2.656
CD8 TEM	0.001	0.464	−0.81	34.477	21.433
CD4 Th2-like	0.006	−0.556	0.698	4.415	7.215
FGF-21	0.008	−0.607	0.58	4.059	5.373
CCL3	0.008	−0.42	0.674	6.303	7.304
CXCL1	0.011	−0.591	0.611	9.23	9.798
CCL4	0.015	−0.478	0.6	6.906	7.691
FGF-23	0.015	−0.525	0.638	0.295	0.676
IL13	0.025	−0.535	0.53	0.504	0.892
ST1A1	0.034	−0.485	0.501	3.272	4.631
PD-L1	0.042	−0.379	0.604	5.363	5.795
CD8 αβ T cells	0.045	0.388	−0.539	8.94	5.296
IL-10RB	0.052	−0.348	0.557	6.951	7.241
CDCP1	0.064	−0.256	0.559	2.417	2.796
TGF-α	0.069	−0.356	0.511	3.765	4.363
TNFSF14	0.085	−0.392	0.454	6.723	7.604
MMP-1	0.092	−0.183	0.452	15.091	15.549
IL-20RA	0.093	−0.265	0.356	1.491	1.67

Data from *n* = 20 patients for serum proteomic (Olink) markers and *n* = 19 patients for cell phenotyping (CyTOF) markers.

### 3.5 Cluster analyses

**Figure 3:**
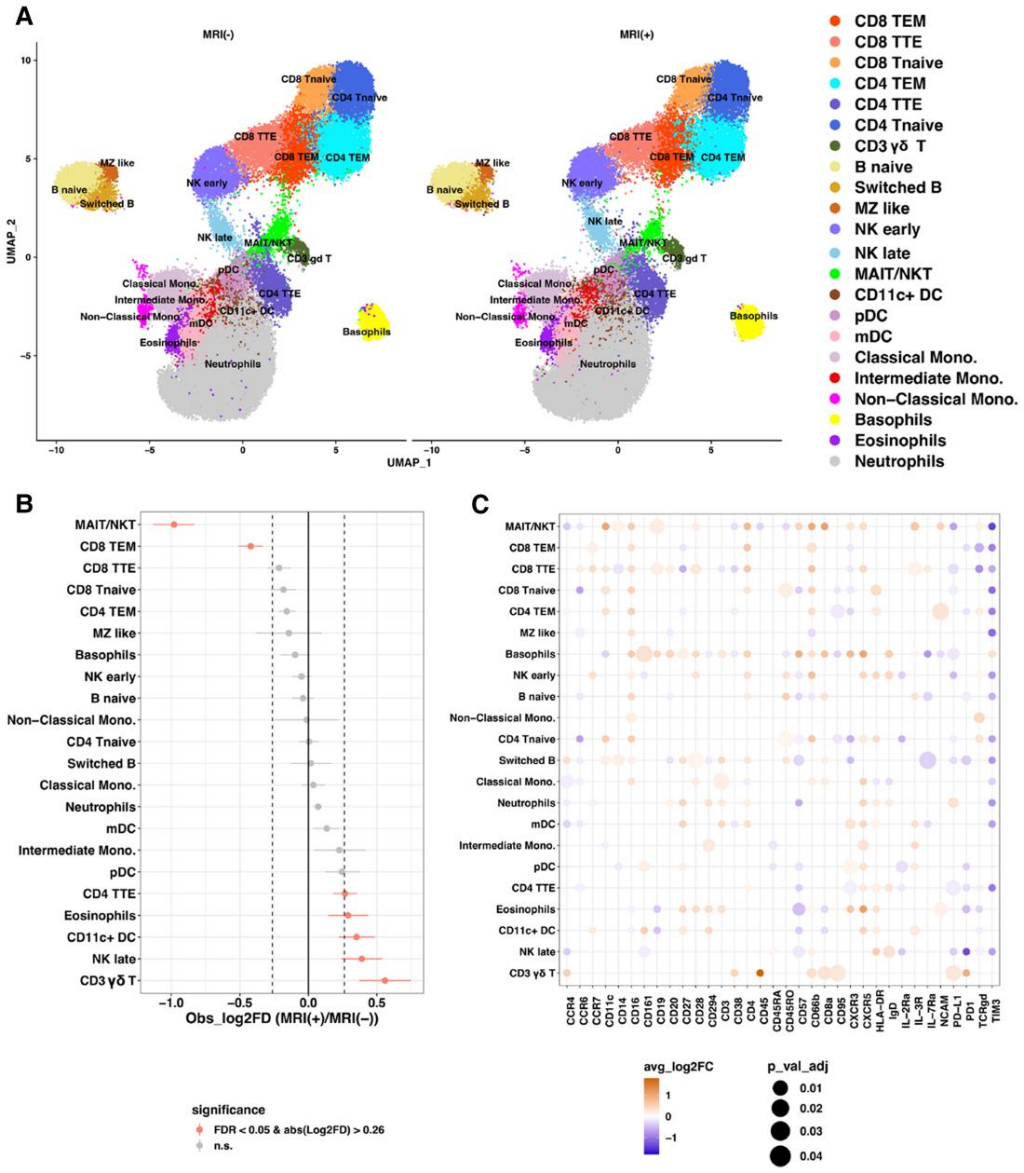
CyTOF cluster analysis for deep immunophenotyping of patients stratified by cardiac MRI. (*A*) UMAP and (*B*) metaplot showing differences in the proportions of cell types from 97013 cells in MRI(+) and MRI(−) patients (*n* = 19) using ‘flowCore’ and Seurat analysis packages. (*C*) Dotplot of average log2 fold change for differential expression of all markers in each cell subset in MRI(+) vs. MRI(−) patients (*n* = 19) using Seurat ‘FindMarkers’ function with ‘Wilcox’ test and selected markers (adj.*P* < 0.05). The dot size represents the significance level, with orange/blue colour scale representing up/down expression for MRI(+) vs. MRI(−), respectively.

Deep immunophenotyping was performed using cluster analysis of the CyTOF data. Twenty-eight clusters were identified, which were annotated based on lineage-specific markers for each cell subset (see Supplementary material online, *Figure S2A* and *B*). CD8 TEM cells were found to be lower in MRI(+) vs. MRI(−) patients, corroborating our previous findings (*Figure 3A* and *B*). Cluster analysis also found MAIT/NKT cells to be lower and CD3γδ, NK, and CD11c^+^ DCs to be higher in MRI(+) vs. MRI(−) patients. Moreover, differential expression of activation and exhaustion markers suggested the presence of an exacerbated adaptive immune response in patients with MRI abnormalities. MRI(+) patients had more highly activated DCs than MRI(−), with increased HLA-DR in mDC and increased CD11c^+^ DC and IL3R in pDC (*Figure 3C*). HLA-DR expression was also increased in CD4^+^ T cells suggesting recent activation.^36^ CD8 T cells had decreased expression of inhibitory receptors (PD1 and TIM3).

**Figure 4:**
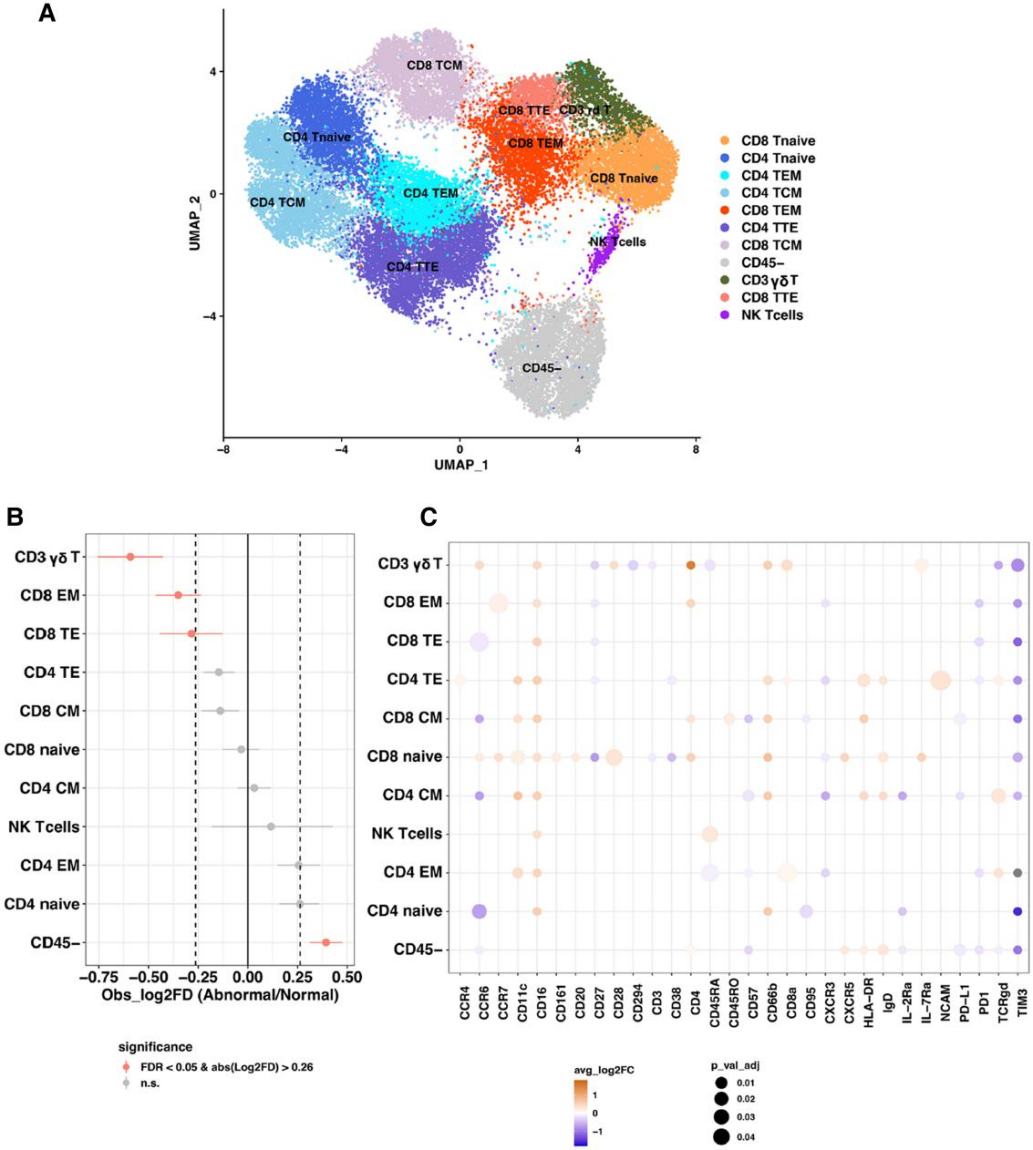
CyTOF re-clustering analysis for CD4/CD8 T cell subsets in patients stratified by cardiac MRI. (*A*) UMAP and (*B*) metaplot showing differences in the proportions of cell types from 97 013 cells in MRI(+) and MRI(−) patients (*n* = 19) using ‘flowCore’ and Seurat analysis packages. (*C*) Dotplot of average log2 fold change for differential expression of all markers in each cell subset in MRI(+) vs. MRI(−) patients (*n* = 19) using Seurat ‘FindMarkers’ function with ‘Wilcox’ test and selected markers (adj.*P* < 0.05). The dot size represents the significance level, with orange/blue colour scale representing up/down expression for MRI(+) vs. MRI(−), respectively.

Further re-clustering of CD3 cells was performed, yielding four clusters of CD4 T cells and four clusters of CD8 T cells (*Figure 4A* and *B*). CD8 TEM cells were again confirmed to be decreased in MRI(+) patients, and these cells had decreased expression of inhibitory receptors (PD1 and TIM3) and decreased expression of CXCR3, a receptor typically associated with interferon gamma (IFNγ) production. UMAP analysis showed that CCR7 was up-regulated in CD8 TEM cells in MRI(+) vs. MRI(−) patients, potentially indicating increased migratory capacity to secondary lymphoid organs and hence lower levels in the blood (*Figure 4C*).^37^ Classical monocytes had decreased expression of chemokine receptors CCR4 and CCR7 despite unaffected cell proportions in blood (*Figure 3C*).

### 3.6 Functional studies

**Figure 5:**
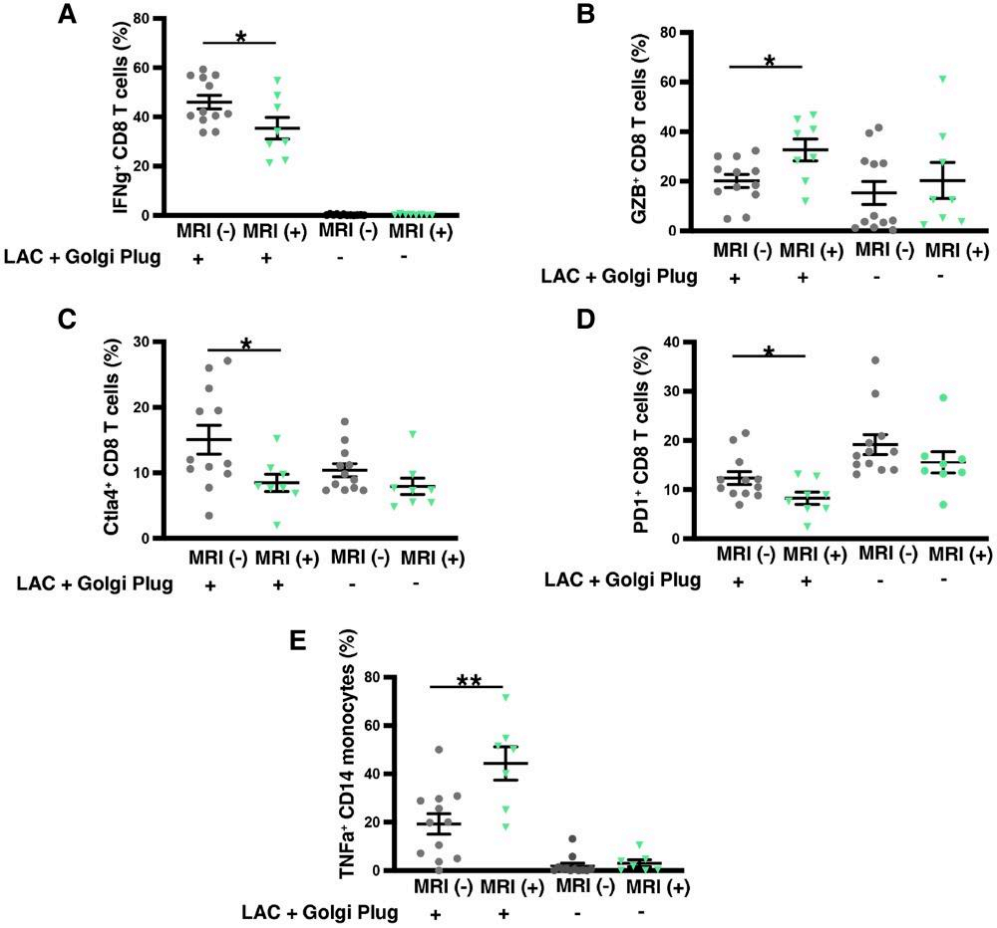
Characterization of CD8 T cells and CD14 monocytes in patients stratified by cardiac MRI. Scatter dot plots comparing proportions of T cells secreting/expressing (*A*) IFNγ, (*B*) GZB, (*C*) CTLA4, and (*D*) PD1 and (*E*) TNF-α secreting CD14 monocytes in MRI(+) and MRI(−) patients (*n* = 20) after stimulation with Leukocyte Activator Cocktail + Golgi Plug compared using Student's *t* test. *P < 0.05; **P < 0.01.

To evaluate the functional capacity of CD8 T αβ cells and CD14^+^ monocytes in patients stratified by MRI, *in vitro* stimulation assays were performed using a Leukocyte Activation Cocktail. There were no frozen PBMCs from one patient. There were lower proportions of IFNγ-producing CD8 T αβ cells after stimulation in samples from MRI(+) vs. MRI(−) patients (*Figure 5A*), but IL-2 secretion was unaffected (data not shown). Interestingly, stimulation of CD8 T αβ cells showed a proportionally greater capacity to produce GZB^+^ in patients with MRI abnormalities (*Figure 5B*), suggesting that these patients had a higher proportion of cytotoxic CD8^+^ T cells despite decreased IFNγ secretion. CTLA4- and PD1-expressing CD8 T αβ cells were lower in MRI(+) than MRI(−) patient samples upon stimulation (*Figure 5C* and *D*), and proportions of unstimulated PD1^+^ CD8 TEM cells were also decreased (*Figure 2E*), again confirming the reduced expression of inhibitory receptors. CD14^+^ monocytes from MRI(+) patients had higher pro-inflammatory TNF-α after stimulation than MRI(−) patients (*Figure 5E*).

### 3.7 Proteomic data

**Figure 6:**
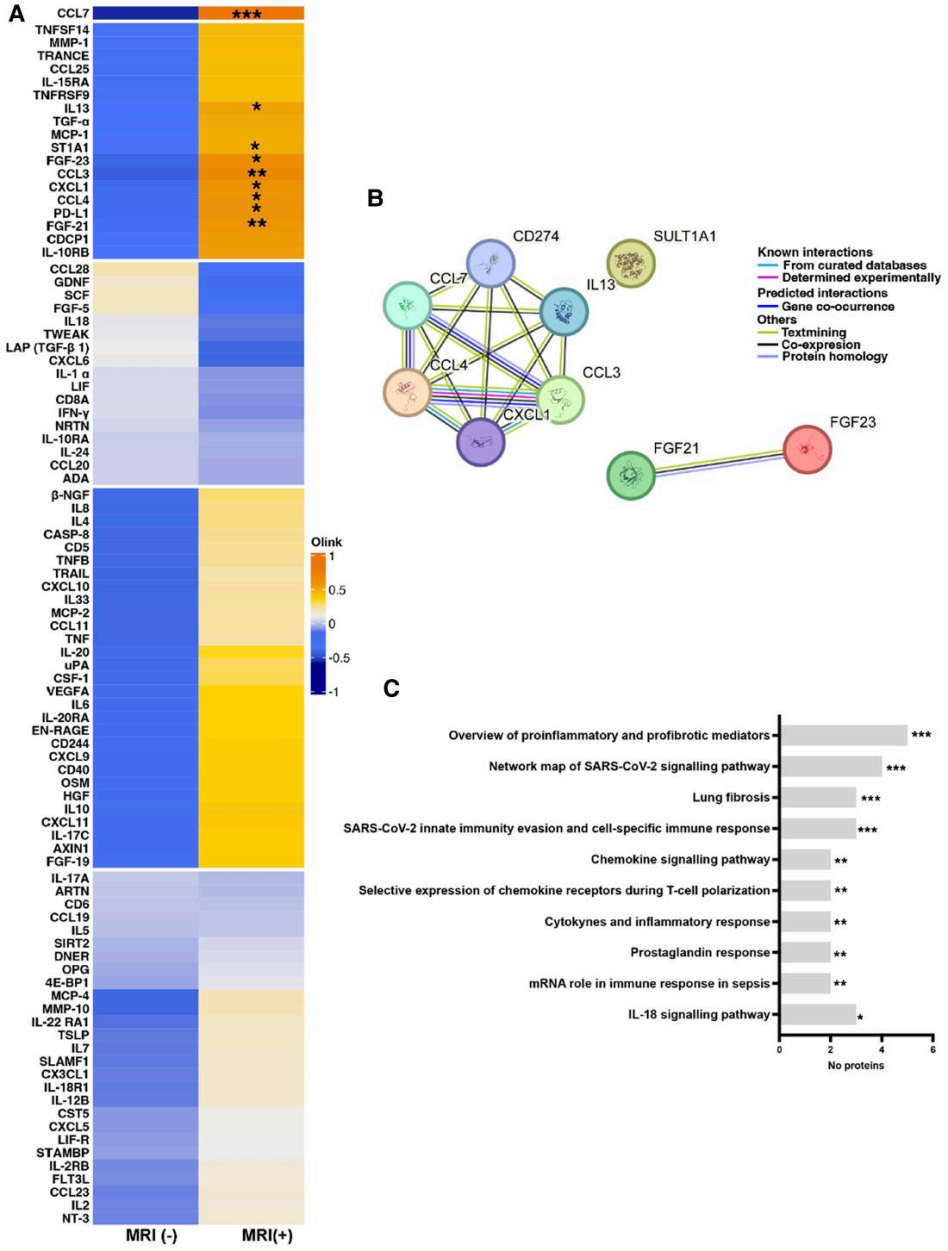
Proteomic inflammatory markers in patients stratified by cardiac MRI. (*A*) Heatmap comparing proteomic inflammatory mediators in patients (*n* = 20) with (+) and without (−) cardiac MRI abnormalities. Transformed data compared using Student's *t* test. (*B*) Functional protein interaction network identified by STRING pathway analysis for the nine differentially expressed proteins (N.B. ST1A1 = SULT1A1; CD274 = PDL1) and (*C*) graph showing selected significantly enriched gene set enrichment analysis pathways. Each bar represents the number of significantly expressed proteins per pathway compared using Fisher's exact test. *P < 0.05; **P < 0.01; ***P < 0.001.

**Figure 7:**
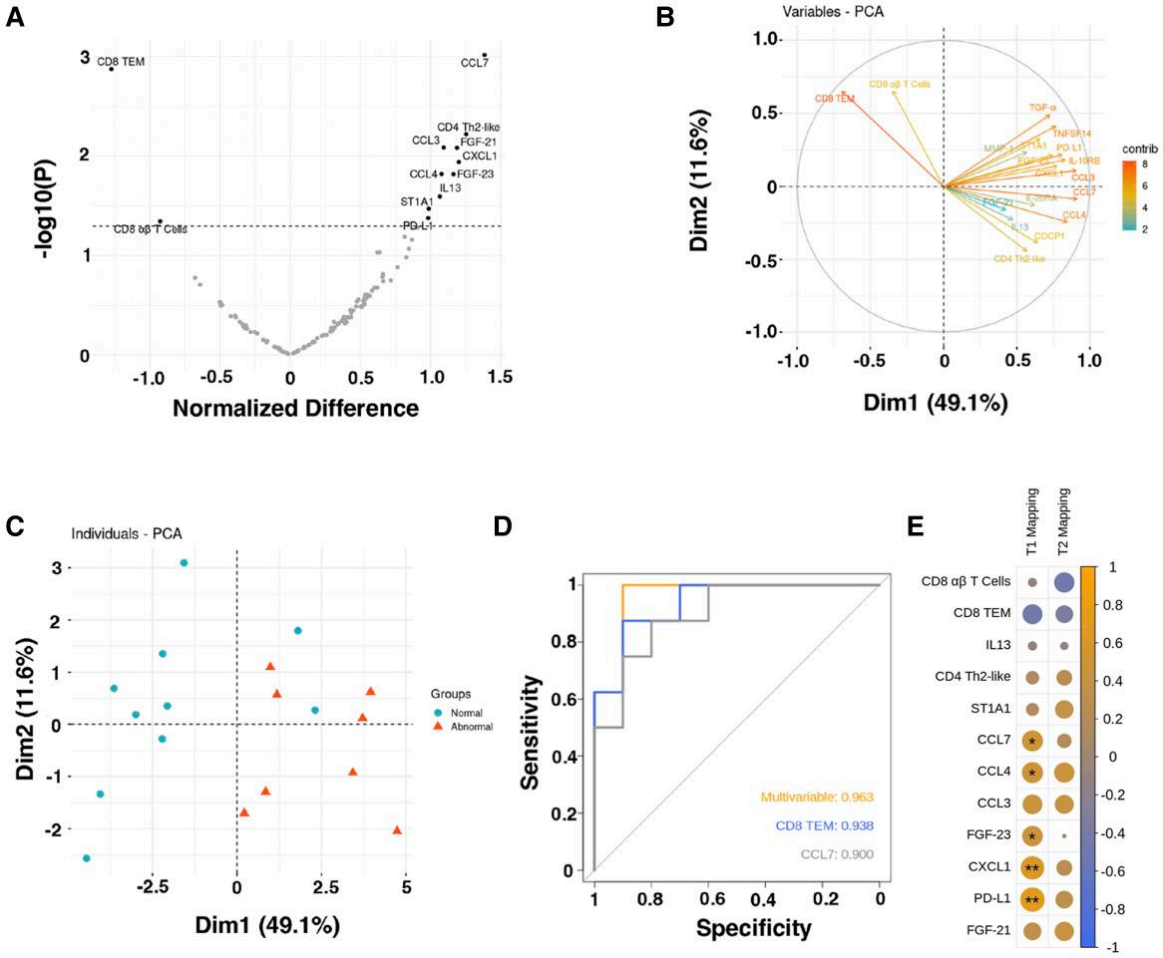
Immune features associated with cardiac MRI abnormalities. (*A*) Volcano plot showing immune features that differed (*P* < 0.05) between participants (*n* = 18) with (+) and without (−) abnormal cardiac MRI findings compared using Student's *t* test; (*B* and *C*) principal component analysis plots showing clustering of candidate features with *P* < 0.1 and the separation of patients (*n* = 18) with MRI(+) and MRI(−) scans based on these features; (*D*) ROC plot showing the AUC for the two strongest predictors of MRI abnormalities (CCL7 and CD8 TEM) identified in the LASSO regression analysis (*n* = 18 patients); (*E*) correlation matrix showing Pearson correlations between the 12 candidate immune markers that differed (*P* < 0.05) between patients (*n* = 18) with (+) and without (−) MRI abnormalities, plus quantitative T1/T2 mapping values on MRI. The colour intensity and size of the circles are proportional to the absolute values of the correlation coefficients. *P < 0.05; **P < 0.01.

Analysis of circulating proteomic immune markers revealed nine differentially expressed proteins between the MRI(+) and MRI(−) groups (*Table 3* and *Figures 6A* and *7A*). There was an insufficient amount of blood taken from one patient for Olink analysis. Markers related to leukocyte chemoattraction were increased in participants with MRI abnormalities compared to those without, including CCL3, CXCL1, and CCL4. Products related to CD4 Th2 responses (IL13), antiviral responses (CCL7), systemic inflammation (FGF-21, FGF-23, and ST1A1), and immune regulation (PD-L1) were also increased more in those with MRI abnormalities than without.

### 3.8 Pathway analysis

To explore potential mechanisms and functions of the nine differentially expressed proteins, unsupervised analysis of protein–protein interactions gene set enrichment analysis (WikiPathways) was also performed using STRING (*Figure 6B* and *C*). These analyses revealed up-regulation of pathways related to inflammation and fibrosis as well as those linked to cell-specific immune responses and evasion of the innate immune response in SARS-CoV-2.

### 3.9 Predictors of COVID-19-related myocardial injury

A clear separation of patients with MRI(+) and MRI(−) scans was observed using principal component analysis to visualize candidate features with *P* < 0.1 (*Figure 7B* and *C*). To identify the most clinically relevant immune markers for predicting MRI-detectable myocardial abnormalities in patients after COVID-19 infection, a LASSO regression analysis was performed, which included all data on circulating immune cell subsets and inflammatory mediators as well as baseline clinical characteristics. CCL7 and CD8 TEM cells were found to be the most important predictors in the statistical model (composite AUC 0.96, 95% confidence interval 0.88 to 1; *Figure 7D*). These predictors remained regardless of whether the analysis included data from all patients or was confined to the long COVID-19 group only with patients enrolled due to raised cTn I or heart failure excluded.

### 3.10 Correlations of immune cell subsets, inflammatory mediators, and imaging

Correlations between differentially expressed immune cell subsets and serum proteins in MRI(+) vs. MRI(−) and quantitative MRI mapping values were assessed (*Figure 7E*). Quantitative T1 mapping values reflective of diffuse interstitial fibrosis and/or oedema were correlated with markers associated with leukocyte chemoattraction (CCL4, CCL7, and CXCL1), immune regulation (PDL1), and fibrosis (FGF-23; Supplementary material online, *Figure S3A–D*). T1 values were measured in myocardial segments with LGE, or the basal septum in patients with no LGE. CCL7 also differed between patients with LGE (+) and those without LGE (−) (Supplementary material online, *Figure S3E*). Of the 12 differential markers, none was associated with T2 mapping values. Although there was an apparent association between CCL7 and LV ejection fraction (%), this finding was driven by the two patients with severe heart failure (data not shown). With only two patients in this category, the study was underpowered to formally assess the relationship with LV function. Correlations between the 12 protein biomarkers and cell fractions are shown in Supplementary material online, *Figure S4*.

## 4. Discussion

Here, for the first time to our knowledge, we show that specific circulating immune cell subsets and inflammatory mediators are associated with clinically detectable cardiac MRI abnormalities in prospectively enrolled patients with cardiovascular signs and symptoms arising after COVID-19 infection (Graphical abstract). Of the 21 individuals studied with clinically suspected cardiac involvement in COVID-19, we observed overt cardiac MRI abnormalities in 9 patients (43%). These patients all had either non-ischaemic patterns of cardiac fibrosis and/or focal myocardial oedema or global ventricular impairment, suggestive of underlying myocarditic processes. We identified important associations between these myocarditis-like MRI abnormalities and altered immune responses, including increased cytotoxic CD8 T cells, CD4 Th2 cells, TNF-α producing monocytes, and several key inflammatory mediators. Overall, the combination of elevated blood CCL7 levels and decreased CD8 TEM cell counts were the most highly predictive markers of abnormal cardiac MRI findings, providing new insight into a potential underlying aetiology of unexplained cardiac symptoms post-COVID-19.

### 4.1 What are the mechanisms of cardiac injury after COVID-19?

The clinical imaging findings of our study are overall in keeping with other reports in the literature where cardiac MRI was performed in patients with suspected cardiac involvement in COVID-19. Indeed, cardiac MRI abnormalities have been reported in up to 61% of individuals in this context, with a higher prevalence of findings consistent with both MI and probable recent myocarditis, than controls.^16^ However, nearly all these studies focused on individuals with cTn elevation in the setting of a hospital admission for COVID-19 infection, with imaging performed in the early convalescence phase.^16–18^ These studies also did not exclude patients with known cardiovascular disease (CVD), nor those treated for acute ischaemic events, and so it is unsurprising that they observed more patients with ischaemic LGE patterns than we did. Importantly, a case-control study of 346 patients without prior heart disease who underwent cardiac MRI scanning a median 9 months after mild COVID-19 infection (that is more comparable to our study population) corroborates our finding of non-ischaemic myocardial scarring suggestive of myocarditis in patients with long COVID-19.^19^ In that study, the 73% of individuals with cardiac symptoms at baseline also had higher T1 mapping values, reflective of diffuse myocardial fibrosis/oedema, and more pericardial involvement than those who were asymptomatic. Moreover, female gender and higher baseline myocardial T1 values predicted the presence of cardiac symptoms at 1-year follow-up. Interestingly, we also found native myocardial T1 values to be an important imaging feature correlated with key circulating immune markers, including CCL4, CCL7, CXCL1, and PDL1. Consistent with this previous study about long COVID-19, our findings also suggest that a proportion of people with persistent cardiac symptoms after COVID-19 infection could have unresolved subclinical myocarditis as evidenced by ongoing myocardial oedema and/or fibrosis detectable by MRI.

### 4.2 How do circulating proteomic markers and immune cells relate to MRI findings?

We found that increased CCL7 levels and decreased CD8 TEM cells, with reduced expression of inhibitory receptors and increased cytotoxic phenotype, were strongly associated with the presence of cardiac MRI abnormalities in patients after COVID-19 infection. CCL7 is an important chemokine that promotes leukocyte recruitment to sites of infection/inflammation.^38^ Based on our findings, it is conceivable that increased circulating CCL7 could be facilitating cytotoxic CD8 T cell recruitment to the heart, with potentially detrimental effects on cardiac remodelling post-COVID-19. High levels of CCL7 have been associated with an overwhelming myocardial inflammatory response to coxsackievirus B3-induced myocarditis in mice,^39^ while the absence of CCL7 has been associated with decreased central nervous system CD8 T cell infiltration during West Nile virus infection.^40^ This hypothesis is further supported by autopsy studies showing myocardial CD8 T cell infiltration in patients who died of COVID-19 infection,^41^ and studies of bronchoalveolar lavage fluid from COVID-19 patients showing high proportions of clonally expanded TEM cells.^42^ Furthermore, CCL7 is among the list of key cytokines/chemokines whose expression levels have been associated with diseased severity and mortality risk in acute COVID-19 infection^21–23,43^ and is an important inflammatory mediator in several other CVDs, including post-infarct myocardial injury, viral myocarditis, and Chagas cardiomyopathy.^44–46^

The finding of increased CCL3 and CCL4 in blood from patients with MRI abnormalities in our study also suggests the possibility of chemokine-dependent CD8 T cell migration. CCL3 and CCL4 are known to enhance CD8 T cell interactions at sites where antigenic CD4 T cell activation occurs.^47^ Circulating CD4 Th2 cells were also found to be increased in patients with abnormal MRIs, along with associated Th2 cytokines (e.g. IL-13 and IL-10RB). We hypothesize that the accumulation of Th2 cells in the blood could reflect decreased migration into the inflamed heart. Switching of Th1 to Th2 polarity is believed to be important during the recovery phase after acute myocarditis, and dysregulation of Th1/Th2 responses has been proposed as a possible cause of post-myocarditic dilated cardiomyopathy.^48^ Other markers that differed in MRI(+) individuals included ST1A1 and FGF-23, which have also been associated with worse outcomes after MI^49^ and asymptomatic COVID-19.^50^ Specifically, FGF-23 is involved in tissue repair, and its elevation could potentially be linked to myocardial fibrosis.

### 4.3 What is the role of CD8 T cells in the heart in COVID-19?

Whether myocardial cytotoxic CD8 T cell accumulation occurs in patients with post-COVID-19 symptoms and myocarditis-like MRI abnormalities, and whether this pathogenic process could be driven by the persistence of SARS-CoV-2-related antigens in the heart, is an intriguing question raised by our study. In an acute viral infection, naïve CD8 T cells that recognize viral antigens become activated, undergo clonal expansion, and differentiate into effector CD8 T cells. These effector CD8 T cells have critical functions in producing inflammatory cytokines and/or directly killing target infected cells via cytotoxic functions. In general, decreased CD8 T cell numbers with decreased activation features and increased exhausted phenotypes have been found in patients with more severe viral infections, suggesting an inability to carry out their protective role.^51^ T cell exhaustion leads to impaired effector function in chronic viral infections, marked by the expression of inhibition receptors (e.g. PD1 and TIM3). In patients with long COVID-19, studies have thus far shown mixed results regarding whether effector CD8 T cells are increased or decreased.^52–54^ Moreover, PD1^+^ SARS-CoV-2-specific CD8 T cells have been shown to be functional, rather than exhausted, in patients with COVID-19 infection.^55^ Further validation would be required to confirm the functional status of PD1^+^ CD8 T cells identified in patients in our study.

The role of cytotoxic CD8 T cells in the pathogenesis of chronic viral and autoimmune myocarditis is well described. We found that patients with abnormal cardiac MRIs had lower circulating CD8 αβ T and CD8 TEM cells with decreased cytokine production and expression of inhibitory receptors, but increased cytotoxicity and migratory capacity. Crucially, stimulation of CD8 T cells led to a greater proportion of GZB^+^-secreting cells in patients with MRI abnormalities than those without, suggesting that these cytotoxic cells could be a potential cause of myocardial injury. Indeed, sustained expression of GZB is an indicator of ongoing T cell-mediated myocardial cell damage, which has been investigated using a fluorogenic nanoprobe in a murine modal of myocarditis.^56^ In that study, dexamethasone reduced GZB activity and the severity of myocardial inflammation/injury. Other factors in support of the hypothesis that these CD8 T cells could be causing cardiac damage include decreased TIM3, PD1, and CTLA4 expression. Decreased expression of these inhibitor receptors could be consistent with T cell-mediated myocardial inflammatory processes, as immune checkpoint inhibitor drugs targeting these proteins are associated with an increased risk of myocarditis.^57,58^ Abnormalities of these immune checkpoint markers could indicate dysfunctional CD8 T cell regulation and thus increased myocarditic potential.^59^ Moreover, it is also possible that the observed increases in CD4 Th2 cells and TNF-α -secreting monocytes could directly contribute to myocardial injury^60,61^ or indirectly by activating cytotoxic CD8 T cells.^62,63^

### 4.4 Study limitations

As the first proof-of-principle study to examine associations between circulating immune markers and cardiac MRI findings in patients after COVID-19 infection, there are several limitations that could impact the generalizability of our findings. These include a relatively small sample size and a lack of histological samples and follow-up imaging. We included participants from several different clinical categories to explore a broader spectrum of COVID-19-related cardiac complications; however, a disproportionate number of patients were enrolled with long COVID-19, and so it was not possible to perform smaller sub-group analyses. Reasons for this occurrence included the desire to exclude patients with cTn elevation due to severe COVID-19 who required steroids (as this would be a confounding effect on immune profiling), and the overall low prevalence of new-onset heart failure attributed to COVID-19. An audit of 207 transthoracic echocardiograms performed at our institution in COVID-19-positive hospitalized patients between March 2020 and March 2021 revealed that only 5% of patients had a LV ejection fraction below 50% that could have potentially been caused by their COVID-19 infection (data not shown). An even lower prevalence of new-onset heart failure among patients affected by COVID-19 has been found in larger retrospective studies.^64^ Although the timing of symptom onset, nature of the MRI findings, and the overall clinical picture support MRI changes being related to COVID-19 infection in this study, pre-existing undiagnosed heart disease cannot be excluded, particularly in the two individuals with new-onset heart failure. Reassuringly, our main conclusions regarding CCL7 and CD8 TEM cells as predictors of cardiac MRI abnormalities remained consistent for both the whole cohort and those with long COVID-19. Lastly, as we assessed proportional immune phenotyping, future work is needed to confirm absolute cell counts.

### 4.5 Conclusions

By integrating advanced clinical imaging and deep immunophenotyping, this unique study reveals novel insights about chronic immune dysregulation associated with non-ischaemic, myocarditis-like COVID-19-related cardiac injury. Further studies are needed to examine the specific roles of CD8 TEM cells, CCL7, and other candidate features, to identify new diagnostic and therapeutic avenues for patients with long-term cardiac sequelae of COVID-19.

**Translational perspective**

Cardiac symptoms are a common feature of long coronavirus disease 2019 (COVID-19) and, more rarely, can be caused by acute COVID-19-related myocarditis or heart failure. However, for many patients with cardiovascular symptoms arising after COVID-19, the underlying cause remains unclear. Diagnostic and therapeutic strategies for cardiac complications of COVID-19 are lacking outside the acute setting, particularly for patients with a mild initial infection. By integrating clinical imaging and deep immunophenotyping in this prospective proof-of-concept study, we show that symptomatic individuals with magnetic resonance imaging evidence of non-ischaemic cardiac injury after COVID-19 have features of dysregulated immune function that could potentially serve as future biomarkers.

## Supplementary Material

cvae159_Supplementary_Data

## Data Availability

Data will be made available upon request after publication of the primary research manuscript.
