# Supplementary material for: Coronavirus disease 2019–related myocardial injury is associated with immune dysregulation in symptomatic patients with cardiac magnetic resonance imaging abnormalities
Source: Cardiovasc Res. 2024 Jul 29;120(14):1752–67. doi: 10.1093/cvr/cvae159 (PMC11587552; doi:10.1093/cvr/cvae159)
Supplement: cvae159_Supplementary_Data [file cvae159_supplementary_data.pdf]

SUPPLEMENTAL MATERIALS

COVID-19 related myocardial injury is associated with immune dysregulation in symptomatic patients with cardiac MRI abnormalities

Andrej Ćorović,[1] Xiaohui Zhao,[1] Yuan Huang,[1] Stephen Newland,[1] Deepa Gopalan,[2] James Harrison,[1] Despina Giakomidi,[1] Shanna Chen,[1] Natalia S Yarkoni,[3] Christopher Wall,[1] Marta Peverelli,[1] Rouchelle Sriranjan,[1] Arianna Gallo,[1] Martin J Graves,[4] Andrew Sage,[1] Paul A Lyons,[5,6] Nyarie Sithole,[7] Martin R Bennett,[1] James HF Rudd,[1] Ziad Mallat,[1] Tian Zhao,[1] Meritxell Nus,*[1] Jason M Tarkin*[1]

[1]Section of Cardiorespiratory Medicine, Department of Medicine, University of Cambridge, Cambridge, UK
[2]Department of Radiology, Cambridge University Hospitals NHS Trust, Cambridge, UK
[3]Cell Phenotyping Hub, Department of Medicine, University of Cambridge, Cambridge, UK
[4]Department of Radiology, University of Cambridge, Cambridge, UK
[5]Cambridge Institute of Therapeutic Immunology and Infectious Disease, Jeffrey Cheah Biomedical Centre, Cambridge Biomedical Campus, Cambridge, UK
[6]Department of Medicine, University of Cambridge, Cambridge, UK
[7]Infectious Diseases, Department of Medicine, University of Cambridge, Cambridge, UK

Supplemental Methods

Inclusion Criteria:

- Patients >18 years old
- Confirmed history of COVID-19 infection **AND** Troponin I elevation >99th percentile of upper reference limit **OR** new-onset heart failure
- Able to give written, informed consent

Exclusion Criteria:
- Women of child-bearing potential not using adequate contraception
- Contra-indication to MRI scanning Contrast allergy or contrast-nephropathy
- Chronic kidney disease (eGFR <30 mL/min/1.73 m^2)
- Previous myocardial infarction or heart failure
- Uncontrolled atrial fibrillation
- Uncontrolled chronic inflammatory disease
- Severe lymphopenia (<0.2 $x10^9$/L)
- Treatment with immunomodulatory therapies within the last month (excluding inhaled or topical steroid therapy)
- Any medical condition, in the opinion of the investigator, that prevents the participant from lying flat during scanning, or from participating in the study

Supplemental Table 1 | Cell phenotyping markers

Cell population	Antibody markers
**Neutrophils**	$CD45^{lo}$, $CD66b^{+}$, $CD16^{+}$, $CD294^{-}$
**Eosinophils**	$CD45^{lo}$, $CD66b^{+}$, $CD16^{-}$, $CD294^{+}$
**B cells**	$CD45^{+}$, $CD66b^{-}$, $CD56^{-}$, CD14-, $CD19^{+}$, $CD3^{-}$
Naïve B cells	$CD45^{+}$, $CD66b^{-}$, $CD56^{-}$, $CD14^{-}$, $CD19^{+}$, $CD3^{-}$, $CD27^{-}$
Total memory B cells	$CD45^{+}$, $CD66b^{-}$, $CD56^{-}$, $CD14^{-}$, $CD19^{+}$, $CD3^{-}$, $CD27^{+}$
Plasmablasts	$CD45^{+}$, $CD66b^{-}$, $CD56^{-}$, $CD14^{-}$, $CD19^{+}$, $CD3^{-}$, $CD27^{+}CD38^{+}$, $CD20^{-}$
**Total Monocytes**	$CD45^{+}$, $CD66b^{-}$, $CD19^{-}$, $CD20^{-}$, $CD3^{-}$, $CD56^{-}$, $CD11c^{+}$, HLA-DR^{+}, $CD14^{+/-}$
Classical monocytes	$CD45^{+}$, $CD66b^{-}$, $CD19^{-}$, $CD20^{-}$, $CD3^{-}$, $CD56^{-}$, $CD11c^{+}$, HLA-DR^{+}, $CD14^{+}$ $CD38^{+}$
Transitional monocytes	$CD45^{+}$, $CD66b^{-}$, $CD19^{-}$, $CD20^{-}$, $CD3^{-}$, $CD56^{-}$, $CD11c^{+}$, HLA-DR^{+}, $CD14^{int}$ $CD38^{lo/-}$
Nonclassical monocytes	$CD45^{+}$, $CD66b^{-}$, $CD19^{-}$, $CD20^{-}$, $CD3^{-}$, $CD56^{-}$, $CD11c^{+}$, HLA-DR^{+}, $CD14^{-}$ $CD38^{-}$
**NKs**	$CD19^{-}$, $CD20^{-}$, $CD3^{-}$, $CD14^{-}$, $CD45RA^{+}$, $CD123^{-}$, $CD45^{+}$, $CD56^{+}$
Early NKs	$CD19^{-}$, $CD20^{-}$, $CD3^{-}$, $CD14^{-}$, $CD45RA^{+}$, $CD123^{-}$, $CD45^{+}$, $CD56^{+}$, $CD57^{-}$
Late NKs	$CD19^{-}$, $CD20^{-}$, $CD3^{-}$, $CD14^{-}$, $CD45RA^{+}$, $CD123^{-}$, $CD45^{+}$, $CD56^{+}$, $CD57^{+}$
**DCs**	$CD3^{-}$, $CD14^{-}$, $CD56^{-}$, HLA-DR^{+}, $CD123^{-}$, $CD11c^{+}$
pDC	$CD3^{-}$, $CD14^{-}$, $CD56^{-}$, HLA-DR^{+}, $CD123^{-}$, $CD11c^{-}$
mD	$CD3^{-}$, $CD14^{-}$, $CD56^{-}$, HLA-DR^{+}, $CD123^{-}$, $CD11c^{+}$ $CD38^{+}$
**CD8αβ T cells**	$CD14^{-}$, $CD11c^{-}$, $CD45^{+}$, $CD3^{+}$, $TCR\gamma\delta^{-}$, $CD4^{-}$, $CD8^{+}$, $CD161^{lo/-}$, $CCR7^{hi}$
CD8 naïve	$CD4^{-}$, $CD8^{+}$, $CD161^{lo/-}$, $CCR7^{hi}$, $CD45RA^{+}$, $CD45RO^{-}$
CD8 central memory	$CD4^{-}$, $CD8^{+}$, $CD161^{lo/-}$, $CCR7^{hi}$, $CD45RA^{-}$, $CD45RO^{+}$

CD8 effector memory	$CD4^-$, $CD8^+$, $CD161^{lo/-}$, $CCR7^{lo/-}$, $CD27^+$
CD8 terminal effector	$CD4^-$, $CD8^+$, $CD161^{lo/-}$, $CCR7^{lo/-}$, $CD27^-$
**CD4αβ T cells**	$CD3^+$, $TCR\gamma\delta^-$, $CD4^+$, $CD8^-$
CD4 naïve	$CD4^+$, $CD8^-$, $CCR7^{hi}$, $CD45RA^+$, $CD45RO^-$
CD4 central memory	$CD4^+$, $CD8^-$, $CCR7^{hi}$, $CD45RA^-$, $CD45RO^+$
CD4 effector memory	$CD4^+$, $CCR7^{lo/-}$, $CD45RA^-$, $CD45RO^+$, $CD27^+$
CD4 terminal effector	$CD4^+$, $CCR7^{lo/-}$, $CD45RA^-$, $CD45RO^+$, $CD27^-$
Tregs	$CD4^+$, $CCR4^+$, $CD45RA^-$, $CD45RO^+$ $CD25^{hi}$, $CD127^{lo/-}$, $CXCR5^-$
Th-1 like	$CD4^+$, $CCR4^-$, $CD45RA^-$, $CD45RO^+$, $CXCR5^-$, $CXCR3^+$, $CCR6^-$
Th-2 like	$CD4^+$, $CCR4^-$, $CD45RA^-$, $CD45RO^+$, $CXCR5^-$, $CXCR3^-$, $CCR6^-$
Th-17 like	$CD4^+$, $CCR4^-$, $CD45RA^-$, $CD45RO^+$, $CXCR5^-$, $CXCR3^-$, $CCR6^+$
**γδ T cells**	$CD3^+$, $TCR\gamma\delta^+$
**MAIT/NKT**	$CD3^+$, $TCR\gamma\delta^-$, $CD4^-$, $CD28^+$, $CD161^{hi}$
**Basophils**	$CD3^-$, $CD56^-$, $HLA\text{-}DR^-$, $CD11c^-$, $CD123^+$, $CD294^+$

Supplemental Table 2 | Antibodies used for functional studies phenotyping

Antibody	Fluorophore	Company
CD45	Sparc Blue 574	Biolegend
CD14	PcP	Biolegend
CD20	BUV395	BD Biosciences
CD3	PE	BD Biosciences
CD4	AF700	Biolegend
CD8	BV605	Biolegend
PD1	PE Fire640	Biolegend
IL2	BV421	BD Biosciences
TNFa	BUV737	Invitrogen
IL13	BV711	BD Biosciences
IFNg	BV750	Biolegend
CTLA4	BUV805	BD Biosciences
CCL7	EF660	Invitrogen
GZB	AF488	Thermo Fisher

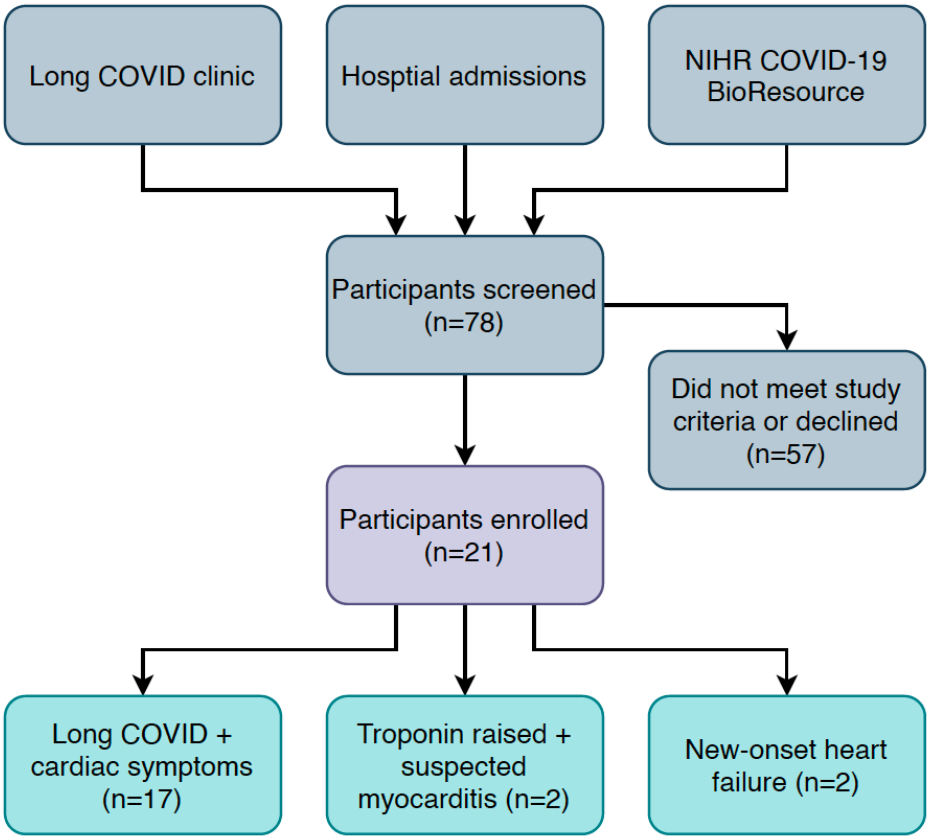

Supplemental Figure 1 | Study flowchart. Summary of participants included in the Multimodality Imaging and Immunophenotyping of COVID-19 related Myocardial Injury (MIIC-MI) study.

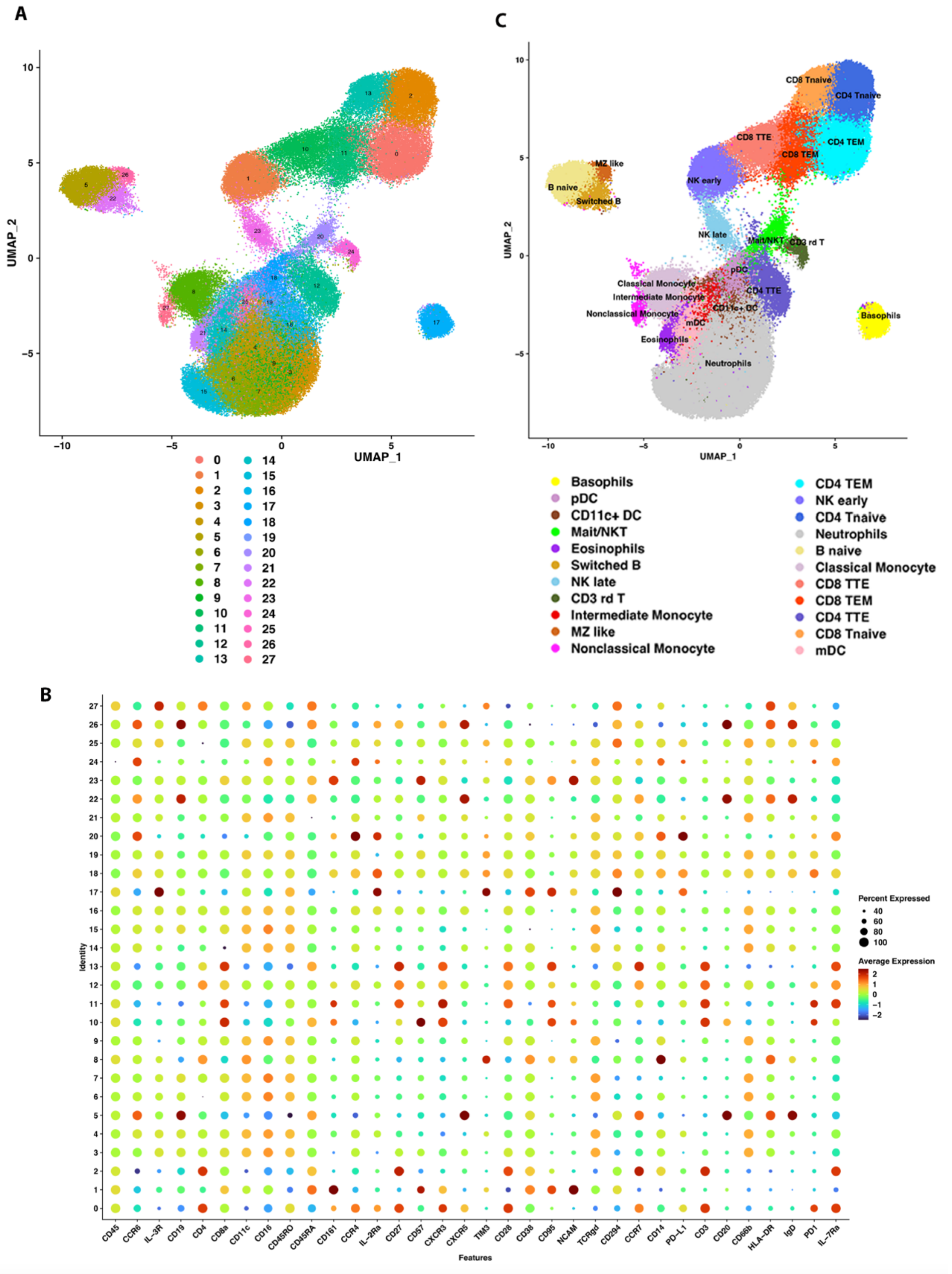

Supplemental Figure 2 | Clustering analysis strategy. (A, C) Automatic clustering analysis using the software packages "flowCore" and Seurat to generate UMAPs; **(B)** Dot plot of the average expression of all markers in each cluster used to annotate immune cell subsets. Analysis of 97013 cells from n=19 patients.

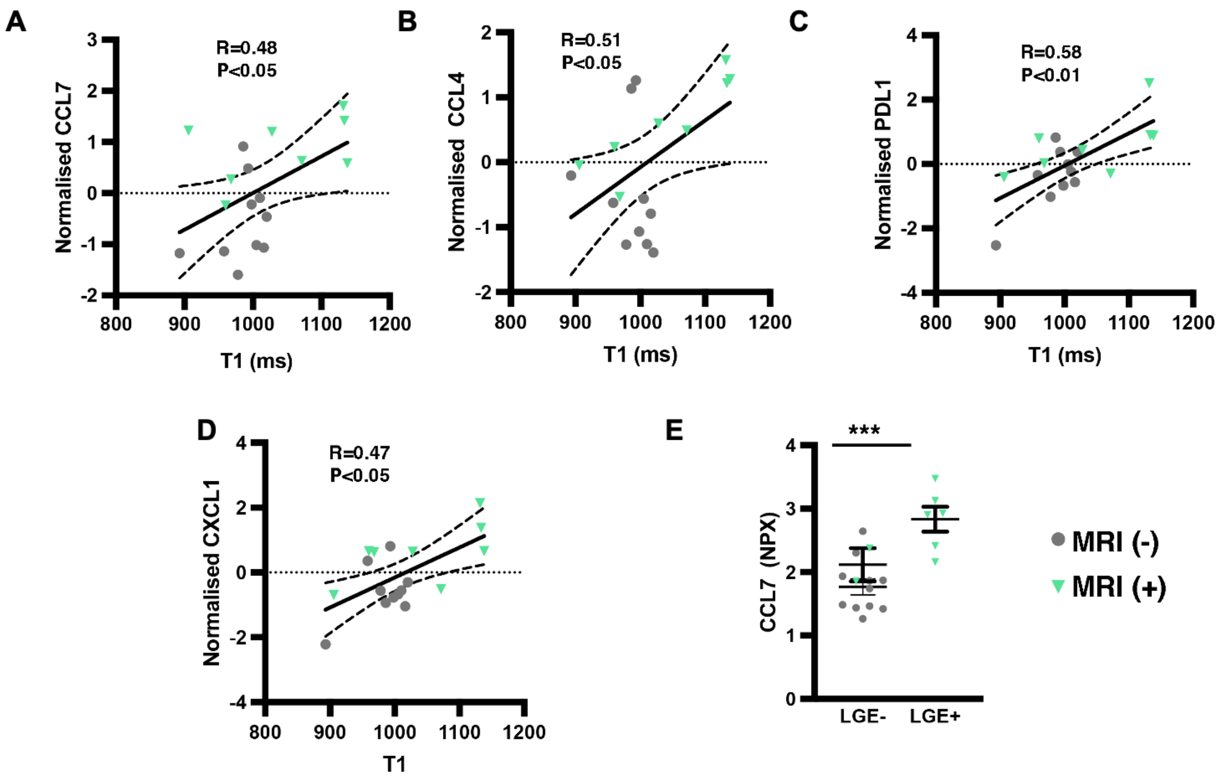

Supplemental Figure 3 | Associations between candidate features and quantitative MRI features. Spearman's correlations of (**A**) CCL7 (**B**) CCL4, (**C**) PDL1 and (**D**) CXCL1 with quantitative T_1-mapping values (a marker of diffuse interstitial fibrosis/oedema) on MRI (n=18 patients); (**E**) scatter dot plot of CCL7 in patients grouped by the presence (+) or absence (-) of myocardial fibrosis defined by late gadolinium enhancement (LGE) MRI (n=19 patients) compared using Student's *t* test. ***P<0.001.

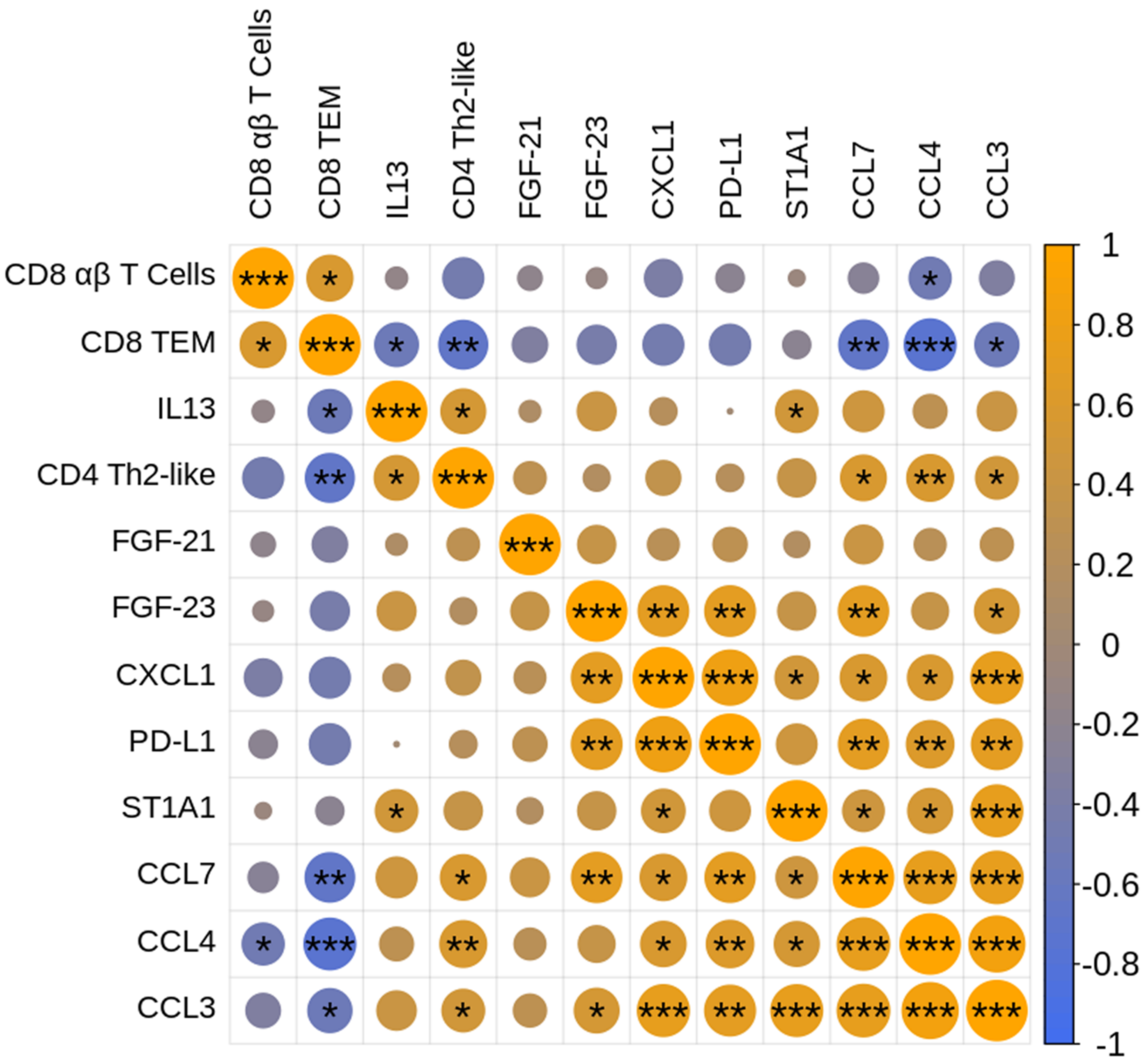

Supplemental Figure 4 | Protein biomarkers vs. cell phenotyping markers. Correlation matrix showing Pearson correlations between proteomic inflammatory biomarkers and cell phenotyping markers that differed (p<0.05) between patients (n=18) with (+) and without (-) MRI abnormalities. The colour intensity and size of the circles are proportional to the absolute values of the correlation coefficients. Plot generated with Pearson correlations in R package "corrplot." *P<0.05, **P<0.01, ***P<0.001.
